# Emigration Effects Induced by Radio Frequency Treatment to Dates Infested by *Carpophilus hemipterus*

**DOI:** 10.3390/insects10090273

**Published:** 2019-08-27

**Authors:** Marzia Cristiana Rosi, Francesco Garbati Pegna, Anita Nencioni, Roberto Guidi, Michele Bicego, Antonio Belcari, Patrizia Sacchetti

**Affiliations:** 1 Department of Agriculture, Food, Environment and Forestry (DAGRI), University of Florence, 50144 Florence, Italy; 2 Stalam, Radio Frequency Equipment, Via dell’Olmo, 7, 36055 Nove (Vicenza), Italy

**Keywords:** sap beetles, RF treatment, emigration, heating rate, physical pest control

## Abstract

The dried fruit beetle (*Carpophilus hemipterus*) is considered a key pest of dates, infesting fruits both in the field and during storage. Control measures against the species rely on the use of chemicals or heat treatments based on sunlight, hot air or radio frequency (RF) applications. Previous investigations that have aimed to define control procedures for a total disinfestation using RF treatments with different exposure durations have shown the typical behavior of adults in some trials, which, under the influence of temperature increases, started to escape from infested fruits. We focused on the application of different RF voltage-time combinations to induce the emigration of *C. hemipterus* adults from dates in order to produce a complete disinfestation. The results showed that the application of 2500 V RF for 8–10 min to infested dates resulted in nearly 100% of adults escaping from fruits, thereby cleaning the commodity with low or no mortality inside and outside the dates. These achievements provide a new strategy for controlling key insect pests of dates that could be applied at an industrial scale, allowing for the quick disinfestation of fruits without affecting the fruit with harmful substances, such as residues from chemical treatments, and without unwanted side effects on date quality.

## 1. Introduction

Dates are available year round, though a large portion of consumption occurs in producing countries where these fruits are eaten as part of the normal diet, especially during the harvest season. Dates can be consumed fresh or dried, depending on the variety [1]. Usually, fresh soft dates can only be stored for no more than a few weeks at very low temperatures (below −18 °C) [2], dry dates can be maintained at room temperature for a long time, and partially dried soft and semi-dry dates are usually stored at a temperature of about 5 °C.

Like other fruits, dates are susceptible to attack by several pests, such as fungi, mites, and insects, either during growth and ripening in the field [3,4] or during postharvest operations and storage [5,6]. Among the insect pests that infest and damage dates, sap beetles (Coleoptera, Nitidulidae) are economically important since they can attack fruits both in the field and during storage [7]. The most common sap beetle that infests dates in almost all producing countries is *Carpophilus hemipterus* (Linnaeus, 1758), a species with worldwide distribution that can also feed on a variety of rotten fruits, and is considered a key pest of stone fruits in Australia [7]. This species is characterized by a noticeable tolerance to high temperatures, which allows its wider distribution in comparison to other sap beetles in warm areas where date palms are cultivated [6]. Like other Nitidulids, *C. hemipterus* adults are attracted by a powerful aggregation pheromone that causes a number of specimens to gather into a few dates [8]. Beetles are good fliers and actively search for ripening fruits in date orchards, as well as fermenting fruits dropped to the soil. Economic damage is caused by both the adults and larvae that feed on the date pulp, which directly determines qualitative and quantitative losses. Aside from the direct damage, beetles can carry microorganisms associated with fermentation processes, which produce aflatoxins harmful to humans [7].

High quality dates should meet grading requirements such as optimal organoleptic hallmarks and the absence of damage or pest residues [9], so postharvest date processing includes disinfestation treatments [10]. Traditionally, prior to storage or packing for sale, dates were disinfested using heat treatments in ovens (60 °C for about 20 min) or by fumigation with methyl bromide or phosphine; more recently, a modified atmosphere with high concentrations of carbon dioxide is being adopted [10,11]. However, these methods all present significant drawbacks, such as possible alterations of organoleptic characteristics due to heat treatments, or health and environmental hazards in the case of fumigation with toxic gases [12]. Another important aspect of these disinfestation methods is that they have to be applied in a sealed environment, which requires batch processing, and, in the case of fumigation or a modified atmosphere, a long treatment time. For example, to use a combination of CO_2_ and phosphine, a minimum of 24 h at 35 °C is required [13], whereas five days or more are necessary when used alone at lower temperatures [14]. As a consequence, these kinds of treatment are not suitable for the fast processing of a large amount of product, as might be necessary for immediate postharvest disinfestation in large plantations.

Recent experiments with the use of electromagnetic radiation, microwaves and radio waves, have demonstrated the possibility of continuous processing for date disinfestation, which would provide a fast, reliable, safe, and environmentally friendly solution to this problem [15]. Radio frequency (RF) treatments have already been applied as a control procedure against several insect pests of stored products, such as cereals [16], dried legumes [17,18], and nuts [19,20,21], on the basis of the differential heating of insects with respect to the infested product [22]. Food materials can store and dissipate electric energy when subjected to an electromagnetic field. The power dissipation is determined by the continuously re-orienting of polarized molecules or ions present in the material to the alternating electric field; these movements convert electromagnetic energy into heat [23,24]. In biological matter, the power absorption and dissipation are influenced by the electric field intensity and by the dielectric properties of the materials: the dielectric constant *ε’* (ability to absorb energy) and the dielectric loss factor *ε”* (ability to dissipate energy as heat) [25]. RF energy, produced through an electromagnetic field generated by two parallel plate electrodes, causes the heating of the product that is irradiated, depending on specific variables related to the material including the water content [23,24,26,27]. RF disinfestation treatments have mainly been developed for dry products, due to the considerable difference between the moisture content of the commodity and that of the insect pest. Given the higher insect water content with respect to the irradiated product, insects treated with RF energy can be heated until death with a shorter application time when compared to traditional heating methods [28]. When RF is applied to foods, in the “general energy balance equation” the terms of the power loss (convection, conduction, etc.) can be considered negligible mainly because of the fast and volumetric heating process or due to the relatively small heat conduction of the material, a linear pattern of the heating process can therefore be hypothesized [29] and as a consequence, the temperature increase mainly depends on the radio frequency power.

Previous studies have highlighted that a six-minute radio frequency (RF) application by heating the dates to approximately 60 °C resulted in 100% mortality of the larvae, pupae, and adults of *C. hemipterus* without any negative effects on the quality of the dates [30,31]. During some four-minute RF tests, the emigration phenomenon was also observed. The exit of *C. hemipterus* adults from dates processed with heat treatment had been previously discovered [32] and applied to date disinfestation [33,34]. In addition to these studies, which reported the effective control and emigration of sap beetle larvae and adults from infested dates, disinfestation has been successful with long lasting heat exposure (two to three hours) and the use of a discontinuous process. RF treatments applied to date disinfestation have shown the possibility of continuously processing a larger quantity of infested dates in a shorter time without affecting the quality of the dates [31].

In this study, we aimed to apply RF irradiation to Deglet-Nour dates infested by *C. hemipterus* adults to develop an effective heating method to induce beetles to exit from the fruit. The ultimate goal was to obtain fruit devoid of dead beetles, which would negatively affect the quality of the dates. We investigated also whether the heating rate had different effects on the adults of *C. hemipterus*. Our main findings showed that low-voltage RF treatments applied for 5–10 min induced a slow heating rate, which allowed the emigration of adults from the infested dates. 

## 2. Materials and Methods

### 2.1. Insects

A small-scale colony of *C. hemipterus*, starting with adults collected from infested stored dates, was maintained at the entomology labs of the Department of Agriculture, Food, Environment and Forestry (DAGRI, University of Florence, Italy) for about 20 generations on an artificial substrate. Beetles were reared in cylindrical plastic containers (Ø 220 mm, height 220 mm) closed with a lid provided with a nylon net for air circulation. An artificial diet provided in Petri dishes (Ø 10 cm) was used as food for both the adults and larvae as well as for the oviposition substrate. The diet was prepared according to previous research procedures [33] and stored in the refrigerator until use. To prevent dehydration and create the most appropriate environment for the beetles, a black plastic shelter was placed over the diet containers, once inserted inside the cages. Weekly rearing procedures involved replacing the exhausted diet with a fresh one, substituting moistened towel paper set inside the rearing cage, and transferring pupae into new cages for adult emergence. Beetles were maintained in a conditioned rearing room (25 ± 3 °C, relative humidity (RH) 45 ± 10% and 14:10 L:D photoperiod).

### 2.2. Dates and Infestation Procedure

The dates used in this study were bought from the local market; these were of top grade quality of the semi-dry Deglet-Nour variety that were packed on branches and produced in Tunisia. The average fruit measurements (100 dates) were as follows: weight 9.93 ± 1.92 g (maximum 6.1 g; minimum 14.1 g); length 39.5 ± 2.99 mm (maximum 45 mm and minimum 29 mm); fruit diameter 18.95 ± 2.48; and the initial moisture content was 19.6 ± 0.76%.

Date infestation was planned by considering the aggregation behavior of *C. hemipterus* adults [8]; to avoid beetles aggregating in a few fruits and obtain a high number of dates containing beetles, insects were placed in contact with a small number of dates inside a container considered the “infestation unit”. Briefly, three dates were inserted in a small polystyrene container (cup-shaped, 8 cm tall, 450 mL volume) together with 15–20 *C. hemipterus* adults. Insects were introduced twice, at two and three weeks before the planned treatment, to guarantee a stable colonization of fruits. A piece of paper towel was placed over the dates to create a proper shelter for the insects. As such, beetles were allowed to settle to replicate the same conditions in naturally infested dates. The *C. hemipterus* used in this experiment were 7–15 days old with a variable number of males and females. The container with 3 dates and 15–20 beetles formed the infestation unit; 60 infestation units (for a total of 180 dates) were prepared before each treatment session. Treatment trials were performed from December 2017 to April 2018, depending on the availability of infested dates.

### 2.3. Experimental Procedures

Experiments were performed in a pilot-scale RF heating system (Stalam S.p.A., Nove, Vicenza, Italy) with a 3.5 kW nominal maximum power and a frequency of 27.12 MHz, as described in previous research [31]. The RF heating system consisted of an irradiation chamber equipped with two parallel electrode plates of 1200 × 800 mm and a supplementary air heating system. A conveyor belt with modifiable speed was mounted between the electrodes. 

Previous results [31] proved that an RF irradiation treatment of six minutes at 5000 V induced a 100% mortality of the *C. hemipterus* larvae, pupae, and adults infesting the dates. This level of mortality was obtained when the maximum temperature reached about 60 °C. On the basis of these findings, and to induce insect emigration, in this study, infested dates were treated with several combinations of RF voltage and time exposure. Seven combinations of electrode voltage and irradiation time were set (Table 1); for the 3500 V irradiation voltage, only one irradiation exposure was tested, whereas for the other three levels of electrode voltage (2500, 3000, and 5000 V), two different exposure times were set.

In all experiments, the RF chamber temperature was maintained at 40 °C using a forced hot air system to improve heating uniformity [35].

To rule out the possible effects of date handling or transport disturbance on insect emigration, control samples (RF0) were also evaluated. Likewise, in the RF heating experiments, control dates were introduced into the RF apparatus for 600 s, which was equivalent to the longest exposure time of the RF voltage-time combinations.

Before each test, naturally infested dates were randomly collected from each infestation unit and inserted individually into small nylon organza drawstring bags (10 × 12 cm) to facilitate the recovery and count of migrated *C. hemipterus* outside the dates. Dates were carefully inserted into the bags in order to not disturb the beetles dwelling inside the date. Nylon bags were laid out in a single layer inside a rectangular polypropylene frame measuring 295 × 365 mm (70 mm in height) with a fine mesh polyester screen attached to the bottom. The frame containing the dates was used like a tray; this was placed on the conveyor belt and removed at the end of each RF irradiation. For the most part, each RF irradiation trial was a replicate with 15 dates taken from five infestation units. The number of dates per trial as well as the number of replicates depended on the availability of infested dates.

For real-time temperature measurements of the internal temperature produced by the RF treatment inside the dates, four fiber-optic probes connected to a four-channel signal conditioner (Reflex, Neoptix Inc., Québec, QC, Canada) were used. Temperature probes were individually inserted into four dates (through the organza bag opening) located at different positions within the frame so that their averaged measurements could reasonably represent the average temperature reached inside the dates. Temperature probes were also inserted into the dates of the control experiments. Before each irradiation trial, the dates were maintained at room temperature in the Stalam factory (Nove, Vicenza, Italy).

The internal temperature of the dates was recorded every 10 s during the irradiation and after the treatment, until the temperature decreased. Temperatures recorded by the four probes were acquired and stored on a laptop (Toshiba Portégé Z30-B-121, Toshiba Europe GMBH, Neuss, Germany). The temperature variations of the dates under different RF conditions were determined and the relationship between the date temperature and time of radiofrequency application for each treatment was evaluated.

### 2.4. Insect Emigration Assessment

Since the dates were naturally infested by *C. carpophilus* adults, the three dates of each infestation unit could hold different numbers of insects; in some cases, the introduced beetles may have gathered together in the same date; however, some dates were not infested at all. For this reason, date infestation was assessed only after the experiments. Since the increasing temperature due to RF treatment could produce insect emigration or insect death, the number of escaped beetles and the number of dead beetles were assessed as described hereafter. Soon after the end of every irradiation test, the nylon bags containing dates were examined directly in the frame. All insects that had escaped from the date and were displaced inside the nylon bag were counted. Possible fugitive insects that had run outside the nylon bag and were wandering on the frame were excluded from the overall computation. Next, each date was quickly dissected and the number of beetles remaining inside the date was also counted. When specimens of *C. carpophilus* were found dead inside the date, they were examined through a stereomicroscope equipped with a fiber-optic illuminator (Leica/Wild M3Z equipped with L2 illuminator; Leica Microsystems, Wetzlar, Germany) to verify whether they had been killed by the RF treatment. Beetles with a shriveled body and rigid appendices were considered dead insects before the irradiation experiment and were not included in the mortality assessment [36].

### 2.5. Statistical Analyses

The main effect of RF application is the temperature increase of the treated product. However, for a better understanding of how insect emigration could be affected by the heating process during the RF voltage-exposure time combinations, the temperature trend recorded during all of the experiments was analyzed.

The linearity of the temperature increase over the RF application time was evaluated and an improved fit was achieved applying a second-order polynomial model (*y = β*_0_ *+ β*_1_ *x − β*_2_ *x*^2^).

To determine whether the relationship between temperature and RF application time was similar for all treatment combinations, the slopes at the initial time (t_i_) were defined.

The slopes of the RF voltage-exposure time combinations were compared using covariance analysis (ANCOVA) and the null hypothesis of equality between slopes was tested with the elapsed heating time as the covariate [37]. Post-hoc comparisons between the slopes based on a *t* statistic were performed, and the significance level was adjusted with the Šidák test [38].

The maximum temperatures reached for each RF experiment were analyzed through the Kruskall-Wallis test (*H*_0_*: θ*_1_ *= θ*_2_ *= θ*_3_ *… = θ*_7_); post-hoc comparisons were performed with Dunn test statistics (*H*_0_*: θ*_1_ *= θ*_2_), and the alpha value was adjusted per the family wise Type I error rate (comparison not directional) [39]. Work with all statistics was conducted using IBM SPSS Statistics for Windows, Version 25.0 (IBM Corp, Armonk, NY, USA).

Concerning insect emigration, the number of insects that escaped outside the dates was compared to the number remaining inside the fruits, per each combination of RF voltage and exposure time, by using a Chi-square Goodness of fit test (*H*_0_ = 1:1). Insect mortality was calculated as the percentage of RF-killed insects with respect to the overall number of insects present in each treatment; this was assessed only in replicates where some beetles were found dead. The insect emigration rate and insect mortality were analyzed using a Chi-square test of independence [40]; when the null hypothesis of equality was rejected, the Marascuilo procedure for pairwise multiple comparisons was applied (*H*_0_*: p*_1_ *= p*_2_ *= … = p*_8_) [41,42]. The Chi-square and the Marascuilo tests were carried out using Excel software (Microsoft Office Professional Plus, 2016, Microsoft Italia, Milano, Italy).

## 3. Results

### 3.1. Heating Profiles

**Figure 1:**
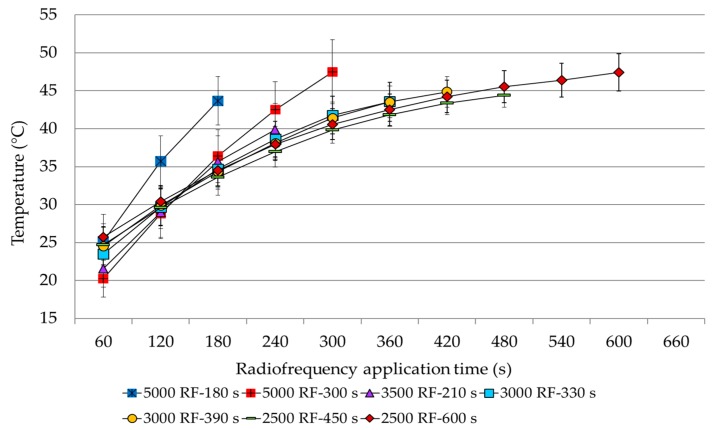
Internal temperature pattern recorded in dates treated with different radio frequency (RF) voltage-time combinations (mean ± Standard Deviation).

The internal temperature of the dates treated with different RF voltage-time combinations increased gradually, as expected, as a function of time with differences in the different experiments. Figure 1 shows the trends in the mean temperatures recorded during RF heating for the selected voltages and time exposures. For each treatment, the regression models were significant (*p* < 0.0001).

During the RF application, the relationship between exposure time and temperature increase showed a noticeable strength of association in all of the RF voltage-time level combinations (Table 2). The determination coefficient (*R*^2^; *p* value < 0.05) value indicates that more than 90% of the variability in temperature increase could be due to the RF irradiation.

The heating process produced different rates of increase among the treatments (Table 2), as shown by the significant differences among slopes at the initial time of exposure (ANCOVA: F(6, 3964) = 6.860; *p* < 0.0001). The fastest temperature increase was observed when the highest voltage was applied; likewise, the heating rate diminished when a lower radio frequency voltage was applied. However, among the radio frequency voltage-time combinations, three groups of analogous values were observed.

The first group included RF voltage-time combinations of 5000 V for 180 s and 300 s, together with the experiment at 3500 V for 210 s. In these treatments, the slope coefficient at the initial time (t_i_) indicated that for every second of irradiation, there was a temperature increase of 0.217 °C, 0.166 °C, and 0.148 °C, respectively. The second group included both the RF voltage-time combination at 3000 V for 330 s and 390 s with a temperature increase of 0.118 °C and 0.100 °C, respectively. Finally, the third group included both RF treatments at 2500 V (applied for 450 and 600 s) with a slope, indicating a corresponding average temperature increases of 0.087 °C and 0.081 °C, respectively, without statistical differences.

The temperature reached in the experiments at the end of the RF process, on average, was always below 50 °C (Table 3, data expressed as the average temperature of the probes ± SD). The highest value was recorded in the experiment with the highest RF voltage of 5000 V for a 300 s irradiation (49.83 ± 4.72 °C); however, this value was not that different from the maximum temperature reached with the lowest RF voltage (RF 2500 V) applied for 600 s (47.94 ± 3.20 °C). In contrast, the lowest maximum temperature was obtained with treatments at RF 3500 V for 210 s (41.31 ± 3.77 °C), which was not statistically different from the value obtained at RF 3000 V for 330 s (44.83 ± 2.69 °C) or at RF 2500 V for 450 s (44.68 ± 1.84 °C). Finally, in the treatments at RF 5000 V for 180 s and those performed at RF 3000 V for 390 s, the maximum temperatures were intermediate values (46.67 ± 2.77 °C and 45.46 ± 2.10 °C, respectively).

### 3.2. Insect Emigration

The number of insects that escaped from the treated dates was always significantly higher than the number of those remaining inside the fruits (Table 4), except in the treatments with the highest radiofrequency voltage (5000 V for 300 s, χ^2^ = 1.922, *p* < 0.166, and 5000 V for 180 s, χ^2^ = 1.064, *p* = 0.302). In contrast, in the control experiment (RF0), the percentage of insects inside the dates (80.56%) was significantly higher than those outside (19.44%) (χ^2^ = 121, *p* < 0.001).

The Chi-square test of independence highlighted significant differences between the number of insects that moved outside the dates or remained inside in the different experiments (*χ*^2^ = 1615.268; df = 7; *p* < 0.0001). Comparisons among the treatments showed that the largest number of insects outside the dates was obtained with a RF voltage below 3500 V, regardless of the irradiation time (Table 4). The percentage of insects outside the dates was 97.63% in the experiments at 3000 V RF for 330 s, 91.39% at RF 3000 V for 390 s, and 95.32% at RF 2500 V for 450 s without any statistical differences among them. The highest levels of emigration occurred at 2500 V for 600 s with 99.10% of adult beetles moving outside the date. In contrast, the lowest proportion of insects that escaped outside the dates was observed when fruits were treated with the RF application at the highest voltage. At RF 5000 V for 300 s, the number of insects that moved outside the dates was 56.86%, which was not significantly different from the values observed with a radio frequency of 5000 V for 180 s (55.32%) or RF of 3500 V for 210 s (70.73%).

### 3.3. Insect Mortality

**Figure 2:**
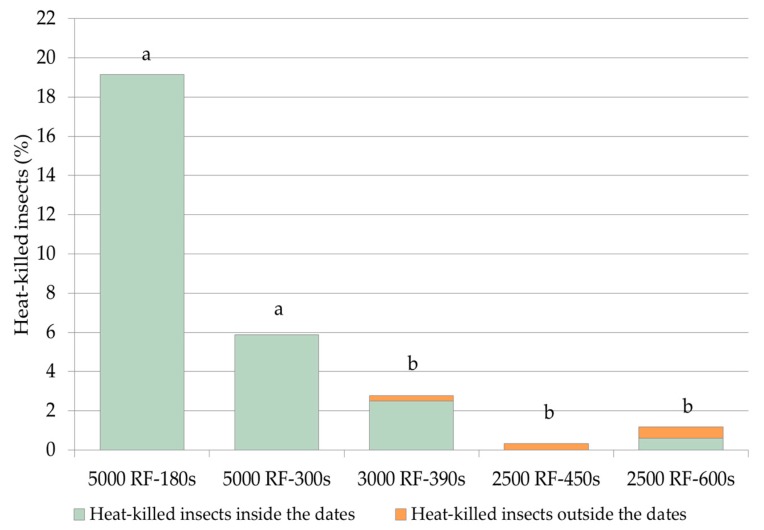
Mortality of *C. hemipterus* adults in the experiments where heat-killed individuals occurred (percentages calculated on the overall number of insects of each experiment). Different letters above the columns indicate significant differences (Chi-square test of independence, χ2 = 88.4732; df = 4; *p* < 0.0001, followed by Marascuilo test to compare proportions; *p* < 0.05).

The insect mortality varied significantly among the experiments (Chi-square test of independence; *χ*^2^: 88.4732; df = 4, *p* < 0.0001) (Figure 2). The highest mortality rate occurred in treatments with the highest voltage (5000 V) with an exposure time of 300 s (19.15%) and 180 s (5.88%). The lowest levels of insect mortality were found at 3000 V for 390 s (3.06%) and 2500 V for 450 s (0.334%) and 600 s (1.14%), without any statistical significance between them. Most of the heat-killed *C. hemipterus* were found inside the dates, whereas few insects were killed by RF treatment after escaping the fruits. A RF of 3000 V applied for 390 s produced mortality inside the dates in 10 insects and only one outside, whereas in RF treatments applied for 600 s, two beetles died internally and two externally. No *C. hemipterus* were found dead in the control experiments and at RF 3500 V for 210 s and RF 3000 V for 330 s.

## 4. Discussion

The findings demonstrate that low-energy RF treatments cause *C. hemipterus* adults to emigrate from infested dates, proving that this disinfestation method could be used to produce insect-free fruit. The low escape rate and null mortality in the control experiments (RF0) indicated that the effects of transportation, handling, and probe insertion were negligible factors inducing emigration; therefore, the results after the RF treatments can be considered a consequence of the RF treatments combined with the exposure time, as highlighted by the coefficient of determination (Table 2).

Heating treatments for the control of food pests have been widely used for quarantine purposes, sanitation, or disinfestation programs. Usually, the temperature of the application is used to obtain the mortality of treated insects due to their intrinsic susceptibility to high temperatures when applied for a proper time [43]. 

Insects, mostly poikilothermic organisms (cold-blooded animals), undergo different changes in their development, metabolism, or physiology when external temperatures exceed their thermal limits. For instance, high temperatures can induce alterations in respiratory physiology and irregularities in the nervous and endocrine systems [44]. The temperature not only affects insect metabolism or physiology, but its variations can also induce behavioral changes to maintain insect body homeostasis. Unfavorable temperatures or sudden thermal variations in the insects’ environment can be perceived by the animal as a forthcoming mortality risk, increased cost of physiological activities, or decline of critical environmental conditions [45]. In response to these changes, insects can adopt strategies to counterbalance the adverse effects of temperatures. Insects can select environments with thermal gradients close to their optimal temperature and can show behavioral strategies where they move away from the heat with reactions depending on the intensity of the stimulus (different kinetic reactions) [45].

Among the different behavioral reactions induced by temperature increase, in a previous work on RF applications to infested dates, the emigration of larvae and adults of the Nitidulid beetle *C. hemipterus* was observed in experiments with low RF energy [31]. As already stated, emigration caused by temperature increase has previously been highlighted [32] and applied to disinfest dates in Israel [33,34]. Infested dates were exposed for two to three hours to hot air produced by different devices to allow the sap beetle larvae to escape from the fruits (and/or kill them eventually). All of these three studies investigated heat treatments as alternative methods to fumigation with methyl bromide. The emigration of Nitidulid larvae and adults had been also achieved in earlier experiments using methyl bromide or physical methods such as modified atmospheres [46,47]. However, none of these control methods reached a complete emigration of the larvae and/or adults from infested dates. The heating treatments required a long exposure time to enable the sap beetles to escape from the fruit. In our experiments, the RF treatments allowed us to achieve a nearly complete disinfestation (95%–99% of adults outside dates) when low RF energy was applied (2500–3000 V) for a short period of time (about 300–600 s). During these treatments, temperature never exceeded 50 °C; therefore, the quality parameters could not be reasonably affected; in previous research, dates treated with RF till the temperature of 60 °C to produce the mortality of the larvae, pupae, and adults of *C. hemipterus* did not show any negative changes in color [31]. Other heat-disinfestation experiments using hot air on Deglet-Nour dates [48] or RF applications on walnuts [49] have demonstrated that a temperature range between 50 and 60 °C does not affect the main quality parameters of dried fruit.

Our results show how heat treatment affected the behavior of *C. hemipterus* adults settled inside dates, causing a quick massive emigration of this pest. As already underlined, in pest disinfestation, heating applications usually have the death of the pest as the primary goal. Heating pest control procedures are based on the knowledge of the kinetics of the thermal death of insect pests that can be experimentally ascertained, and then applied to build models that are useful for the development of specific treatment protocols as well as to predict the mortality of insect species (thermal death time) when subjected to heating in isothermal or non-isothermal conditions [50,51,52,53]. For several insect species, the insect’s susceptibility and sensitivity to temperature changes have been defined [29,54,55]. However, the effects of heating on the behavior of stored product pests are less known, to some extent, and it is possible that the emigration phenomenon could be observed in other species, like the movements reported for the larvae of *Cryptolestes ferrugineus* [56].

In our experiments, we also recorded the internal temperature in dates irradiated with different RF voltage-exposure time combinations. The resulting heat patterns were similar to the temperature pattern displayed in other heat treatments, such as chestnuts treated with 6 kW RF energy [57] or in-shell walnuts irradiated with a 12 kW treatment [58], notwithstanding the different irradiation parameters and purposes. In our opinion, insect emigration was induced by the slow and progressive increase in the temperature of the dates treated with lower RF energy (2500 and 3000 V), which allowed the Nitidulid adults to escape from fruits with the minimal mortality of adults both inside and outside the fruits. The experiments conducted with higher RF energy (5000 and 3500 V) caused a more sudden temperature increase, producing a certain percentage of mortality, similar to previous heating treatments using an oven [34] or in RF applications set at 5000 V [31]. However, the insect mortality percentages recorded in these three RF voltage level-exposure time combinations were different, and for the combinations of 3500 V RF applied for 210 s, no beetles were killed. We observed that applications with the same RF energy of 5000 V and applied for 180 s or 300 s had different starting temperatures (20.5 ± 1.5 °C and 17.96 ± 0.95 °C, respectively, in the two experiment sets) with similar heating rates, producing approximately the same level of beetle emigration with a lower mortality inside the date when the exposure time was longer. This result may be due to the different starting temperatures of the treated dates (see the Y-intercept on Table 2), since the temperature of the material affects the dielectric properties, and consequently the heating rate [24,25]. It is likely that disinfestation protocols should consider the start temperature of the material, depending on the main goal of the process.

During RF treatments aimed at promoting emigration behavior, in addition to the thermal shock caused by a rapid temperature increase (produced by high RF energy) [24], the temperature level that has been proven to be lethal to the targeted insect species must be avoided. A temperature of 60 °C was considered deadly for *C. hemipterus* [31,59]; therefore, in this study, a lower precautionary limit of 50 °C was set to prevent insect mortality inside the dates. However, even in treatments with the lowest RF energy (i.e., 2500 V), when the temperature approached this threshold, some insects were killed (the maximum temperature of 47.94 ± 3.20 °C killed two *C. hemipterus* adults inside the dates). As a consequence, the final temperature reached during heating treatments should also be considered [24]. After the beetles emigrated outside the dates, they could have been easily killed by the RF irradiation. One pioneer study proved that stored grain insect pests are more susceptible to RF effects when insects were irradiated outside the grain kernels, showing a higher mortality than the mortality of insects inside the kernel [24,60]. However, this last RF procedure should be further investigated due to the different susceptibility of each insect species.

To plan postharvest processing for Deglet-Nour dates that includes date disinfestation aimed at inducing *C. carpophilus* emigration, the RF unit needs to be set properly; at the beginning of the treatment, dates should be at a temperature of 20–22 °C and arranged in a single layer on the conveyor belt. In this way, dates can be irradiated for 600 s with a low RF voltage, much like in our experiment. Finally, the adult beetles can be removed from the fruits by blowing them with a fan and/or collecting them with an aspirator device.

## 5. Conclusions

This research allowed us to deepen previous observations of the behavior of *C. hemipterus* adults when subjected to temperature increase produced by RF irradiation. The emigration of the Nitidulid adults appeared to be induced by both the temperature level and the rate of temperature increase. The current findings show that low voltage RF applications can be used to induce sap beetles to escape from infested dates, thus producing clean fruit. In particular, by applying 2500 V RF for 8–10 min to infested dates, an effective disinfestation of the commodity can be obtained, with low mortality inside and outside the dates.

In conclusion, RF technology can be used in date postharvest processing as a disinfestation method that appears to be more convenient than other current procedures that are usually based on the application of chemicals, resulting in a more economically and environmentally friendly procedure. Further research on the effects of RF treatments on the emigration phenomenon of larvae is needed.

RF irradiation has been proven to be a reliable disinfestation method for inducing the emigration of *C. hemipterus* in infested dates; this technique could also be extended to other commodities and insect pests through specific studies.

## Figures and Tables

**Table 1:** Experimental layout of radio frequency time-voltage combinations.

Experiment Name	Irradiation Voltage (V)	Exposure Time (s)	Replicates (*n*)	Dates (*n*)	Insects (*n*)
RF0	0	600	6	90	324
5000 RF-180s	RF 5000 V	180	2	30	94
5000 RF-300s	RF 5000 V	300	2	30	102
3500 RF-210s	RF 3500 V	210	3	45	82
3000 RF-330s	RF 3000 V	330	6	90	253
3000 RF-390s	RF 3000 V	390	6	99	360
2500 RF-450s	RF 2500 V	450	6	90	299
2500 RF-600s	RF 2500 V	600	6	84	335

**Table 2:** Estimates of the polynomial regressions parameters of the relationship between temperature (°C) and RF application time (s) in different RF experiments.

Experiment	Model	R²	SEE	Parameters ± SE	t	Slope (t_i_)
5000 RF-180s	*β*_0_	0.94	2.03	18.087 ± 0.6156	29.378	0.217 a
(*n* = 126)	*β*_1_			0.2179 ± 0.0149	14.608	
	*β*_2_			−0.0003 ± 0.0001	−4.345	
5000 RF-300s	*β*_0_	0.92	2.88	14.8638 ± 0.6436	23.095	0.166 a
(*n* = 210)	*β*_1_			0.1631 ± 0.0096	17.038	
	*β*_2_			−0.0002 ± 0.0000	−5.347	
3500 RF-210s	*β*_0_	0.87	2.62	17.2123 ± 0.5488	31.363	0.148 a
(*n* = 252)	*β*_1_			0.1478 ± 0.0115	12.867	
	*β*_2_			−0.0002 ± 0.0001	−3.066	
3000 RF-330s	*β*_0_	0.92	2.04	19.6108 ± 0.2372	82.672	0.118 b
(*n* = 741)	*β*_1_			0.1184 ± 0.0032	36.809	
	*β*_2_			−0.0001 ± 0.0000	−14.888	
3000 RF-390s	*β*_0_	0.93	1.99	20.5970 ± 0.2310	89.178	0.100 b,c
(*n* = 759)	*β*_1_			0.1005 ± 0.0027	37.759	
	*β*_2_			−0.0001 ± 0.0000	−14.925	
2500 RF-450s	*β*_0_	0.92	1.92	21.2550 ± 0.2410	88.186	0.087 c
(*n* = 630)	*β*_1_			0.0868 ± 0.0024	35.911	
	*β*_2_			−0.0001 ± 0.0000	−15.663	
2500 RF-600s	*β*_0_	0.93	1.95	22.8967 ± 0.1709	133.956	0.081 c
(*n* = 1260)	*β*_1_			0.0814 ± 0.0013	62.921	
	*β*_2_			−0.0001 ± 0.0000	−33.752	

Polynomial regression analysis with temperature as the dependent variable; *n* = total temperature measurements; *R*^2^ = coefficient of determination; SEE = Standard Error of the Estimate; SE = Standard Error; Slope (t_i_) = slope at the initial time of the RF application. *β*_0_ = Y-intercept, *β*_1_ = RF application time; *β*_2_ = (RF application time)^2^. All coefficients were significant at *p* < 0.0001. Different letters in the Slope (t_i_) column indicate significant differences (*p* < 0.05) between the RF voltage-exposure time combinations (pairwise comparisons of the Šidák test).

**Table 3:** Comparison between the maximum temperatures (mean ± Standard Deviation) of the dates at the end of RF heating with different combinations of voltage and exposure time (Kruskal-Wallis test: *N* = number of replicates; H (6, *N* = 108) = 34.41, *p* < 0.0001).

Experiment	*N*	Temperature °C (Mean ± SD) ^1^	
5000 RF-180	7	46.67 ± 2.77	a,b
5000 RF-300	7	49.83 ± 4.72	a
3500 RF-210	10	41.31 ± 3.77	b
3000 RF-330	23	44.83 ± 2.69	b
3000 RF-390	22	45.46 ± 2.10	a,b
2500 RF-450	16	44.68 ± 1.84	b
2500 RF-600	23	47.94 ± 3.20	a

^1^ Different letters in the temperature values indicate significant differences (*p* < 0.05) between maximum temperatures recorded in the experiments. Post-hoc comparisons were performed with Dunn test statistics, and the alpha value was adjusted per the family wise Type I error rate.

**Table 4:** Comparison between the *Carpophilus hemipterus* adults that emigrated outside the dates and those remaining inside in each experiment (difference to 100) (Goodness of fit, Chi-square test 1:1) and comparisons among percentages (Marascuilo test) of *C. hemipterus* adults that moved outside the dates in all the experiments (percentages calculated on the overall number of insects in each experiment). Numbers of *C. hemipterus* include alive and thermal treatment-killed beetles.

Experiment	Beetles Outside the Dates (%)	*χ* ^2^	*p*
RF 0	19.44 a	121.00	<0.001
5000 RF-180 s	55.32 b	1.06	0.302
5000 RF-300 s	56.86 b	1.92	0.166
3500 RF-210 s	70.73 b	14.10	<0.001
3000 RF-330 s	97.63 c,d	229.57	<0.001
3000 RF-390 s	91.39 c	246.68	<0.001
2500 RF-450 s	95.32 c,d	245.62	<0.001
2500 RF-600 s	99.10 d	323.11	<0.001

Different letters in the Beetles outside the dates (%) column indicate significant differences in the pair wise comparisons (Chi-square test of independence, χ^2^ = 1615.268; df = 7; *p* < 0.0001, followed by Marascuilo test to compare proportions); χ^2^ and *p* columns refer to comparisons within the line (Goodness of fit, Chi-square test 1:1).
